# Disseminated Mucormycosis in Immunocompromised Children: Are New Antifungal Agents Making a Difference? A Multicenter Retrospective Study

**DOI:** 10.3390/jof7030165

**Published:** 2021-02-25

**Authors:** Sarah Elitzur, Salvador Fischer, Nira Arad-Cohen, Assaf Barg, Miriam Ben-Harosh, Dana Danino, Ronit Elhasid, Aharon Gefen, Gil Gilad, Itzhak Levy, Yael Shachor-Meyouhas, Sigal Weinreb, Shai Izraeli, Shlomit Barzilai-Birenboim

**Affiliations:** 1 The Rina Zaizov Division of Pediatric Hematology-Oncology, Schneider Children’s Medical Center, 4920235 Petah Tikva, Israel; salvadorf@clalit.org.il (S.F.); gilgi@clalit.org.il (G.G.); sizraeli@gmail.com (S.I.); shlombiren@gmail.com (S.B.-B.); 2 Sackler Faculty of Medicine, Tel Aviv University, 6997801 Tel Aviv, Israel; assaf.barg@sheba.health.gov.il (A.B.); ronite@tlvmc.gov.il (R.E.); Itzhakl@clalit.org.il (I.L.); 3 Pediatric Hematology-Oncology Department, Ruth Rappaport Children’s Hospital, Rambam Health Care Campus, 3109601 Haifa, Israel; n_arad-cohen@rambam.health.gov.il (N.A.-C.); r_gefen@rambam.health.gov.il (A.G.); 4 Rappaport Faculty of Medicine, Technion-Israel Institute of Technology, 3200003 Haifa, Israel; y_shahor@rambam.health.gov.il; 5 Division of Pediatric Hematology, Oncology and BMT, The Edmond and Lily Safra Children’s Hospital, Sheba Medical Center, 5262161 Ramat Gan, Israel; 6 Department of Pediatric Hematology-Oncology, Soroka Medical Center, Ben Gurion University, 8489501 Beer Sheva, Israel; MiriBH@clalit.org.il; 7 Pediatric Infectious Disease Unit Soroka Medical Center, Ben Gurion University, 8489501 Beer Sheva, Israel; danadanino@hotmail.com; 8 Department of Pediatric Hemato-Oncology, Sourasky Medical Center, 6423906 Tel Aviv, Israel; 9 Pediatric Infectious Disease Unit, Schneider Children’s Medical Center, 4920235 Petah Tikva, Israel; 10 Pediatric Infectious Disease Unit, Ruth Rappaport Children’s Hospital, Rambam Health Care Campus, 3109601 Haifa, Israel; 11 Pediatric Hematology-Oncology, Hadassah Hebrew University Medical Center, 9112000 Jerusalem, Israel; sigalv@hadassah.org.il

**Keywords:** children, leukemia, immunocompromised, mucormycosis, invasive fungal infections, antifungal agents, pediatric hematology oncology

## Abstract

Background: Mucormycosis is a life-threatening infection with a tendency for angioinvasion that may lead to progressive dissemination. Disseminated mucormycosis, defined as the involvement of two or more non-contiguous sites, is rare in children, and data concerning its management and outcome are scarce. The aim of this study was to assess the contemporary management strategies and outcomes of disseminated mucormycosis in the pediatric population. Methods: We conducted a retrospective search in six large tertiary medical centers for all cases of disseminated mucormycosis that occurred between 2009–2020 in patients aged 1–20 years. Results: Twelve cases were identified. Underlying conditions included hematological malignancies (*n* = 10), solid tumor (post-autologous hematopoietic stem cell transplantations; *n* = 1), and solid organ (liver) transplantation (*n* = 1). In all cases, amphotericin B formulations were administered as first-line therapy; in eight cases, they were also administered in combination with an echinocandin or triazole. Seven patients underwent surgical debridement procedures. The six-week mortality was 58%. Among the patients diagnosed between 2009–2015, one of the six survived, and of those diagnosed between 2016–2020, four of the six were salvaged. Conclusions: Disseminated mucormycosis is a life-threatening and often fatal disease, and improved diagnostic and therapeutic strategies are needed. Nevertheless, in this population-based study, five patients (42%) were salvaged through combined liposomal amphotericin/triazole treatment and extensive surgical interventions.

## 1. Introduction

Mucormycosis is a life-threatening infection in immunocompromised children, caused by fungi of the order Mucorales. It is characterized by a rapid clinical course, with a tendency for angioinvasion that may lead to progressive dissemination, tissue infarction, and necrosis [1]. Several clinical entities of mucormycosis have been described, including rhino-orbito-cerebral, cutaneous, pulmonary, and gastrointestinal patterns of disease [2,3,4,5]. All of these may potentially evolve into disseminated disease, defined as the involvement of two or more non-contiguous sites. Disseminated mucormycosis is a devastating clinical entity, carrying unacceptable mortality rates, reportedly higher than 90% [6].

Rapid diagnosis, prompt initiation of antifungal therapy, reversal of immunosuppression, and surgical debridement are cornerstones of mucormycosis management [7,8,9]. Because of its rarity and heterogeneity, there are a lack of data from prospective randomized clinical trials to guide the management of mucormycosis. Specific data regarding contemporary outcomes and management strategies of disseminated mucormycosis are even more difficult to elucidate, as these patients are usually the subject of isolated case reports [10,11,12,13,14,15,16] or constitute small subsets in larger studies. Data are even rarer in the pediatric population [3,4]. When treating a patient with disseminated mucormycosis, clinicians are faced with complex decisions, such as the choice of therapeutic agents and the performance of major, potentially mutilating, surgical procedures in critically ill patients.

In this multicenter retrospective study, our aim was to assess the management strategies and outcomes of all children with disseminated mucormycosis diagnosed in six large tertiary medical centers in Israel during the study period.

## 2. Materials and Methods

### 2.1. Study Design

We conducted a retrospective search for cases of disseminated mucormycosis in six large tertiary medical centers. We included all children in these hospitals, which encompassed all six pediatric hematology–oncology departments in Israel. In each center, the medical records, microbiology databases, and pathology information systems were searched for all cases of disseminated mucormycosis that occurred between 1 April 2009 and 1 May 2020 in patients aged 1–20 years. 

Mucormycosis was categorized as proven, probable, or possible, based on the definitions of the European Organization for Research and Treatment of Cancer/Mycoses Study Group (EORTC/MSG) for invasive fungal disease [17]. Mucormycosis was considered disseminated if it involved two or more non-contiguous sites. Time of mucormycosis diagnosis was defined as the date on which the diagnostic procedure was performed. The primary outcome of the study was the 6-week case fatality rate from the day of mucormycosis diagnosis.

Five patients had been included in a previous general study [3], and one in a previous case-report [18], but have been presented here in greater detail and with a longer follow-up.

### 2.2. Diagnostic Methods

Identification of the cultured Mucorales was performed at various institutional microbiology laboratories using standard phenotypic methods. When possible, fresh specimens were sent to a central reference laboratory (Clinical Microbiology Laboratory, Rambam Health Care Campus, Haifa, Israel) for DNA sequencing and analysis. DNA was extracted from clinical samples using the QIAamp DNA Mini Kit (Qiagen, Valencia, CA, USA), and was amplified using a semi nested polymerase chain reaction (PCR) assay targeting specific zygomycete regions at the 18S rDNA, as well as a fungal-broad range PCR reaction targeting the 28S rDNA. The PCR products were separated by electrophoresis in ethidium bromide stained 2% agarose gels, then sequenced on a 3130 Genetic Analyzer capillary electrophoresis DNA sequencer (Applied Biosystems, Carlsbad, CA, USA) and analyzed using the Basic Local Alignment Search Tool (BLAST). 

Antifungal susceptibility testing was performed at the National Mycology Reference Laboratory (Sourasky Medical Center, Tel Aviv, Israel). The minimal inhibitory concentrations (MICs) of the antifungal agents for each isolate were determined using broth microdilution.

## 3. Results

### 3.1. Patient Characteristics

Twelve pediatric cases of disseminated mucormycosis, occurring between 2009–2020, were identified for inclusion in the study. The median age of the study group was 13.5 years (range 8.9–19.2 years). Ten patients had an underlying hematological malignancy (acute lymphoblastic leukemia (ALL), *n* = 5; acute myeloid leukemia (AML), *n* = 4; and mature B-cell (Burkitt) leukemia, *n* = 1), one had been diagnosed with a solid tumor (metastatic medulloblastoma), and one patient was a solid organ (liver) transplant recipient. Two patients underwent hematopoietic stem cell transplantation (HSCT) prior to mucormycosis diagnosis. One patient, with relapsed ALL, was an allogeneic HSCT recipient, and the other, with medulloblastoma, was an autologous HSCT recipient. Nine patients were diagnosed during the course of their chemotherapeutic regimens, as follows: five during the first month of remission induction and four after consolidation chemotherapy courses. None of the patients had a refractory, uncontrolled underlying malignancy. The median duration of neutropenia prior to mucormycosis diagnosis was 12.5 days (range 0–35 days). Table 1 summarizes the patient characteristics.

### 3.2. Clinical Presentation and Pathogens

Ten of the twelve patients presented with prolonged fever. Other presenting symptoms included abdominal pain, chest pain and dyspnea, convulsions, hemiparesis, and obtundation. Anatomical sites involved included the liver (*n* = 8), bowel (*n* = 6), lung (*n* = 6), brain (*n* = 4), and skin (*n* = 2). Three cases were presumably of a sinopulmonary origin, four were from the gastrointestinal tract, and one was from a liver allograft; in the four other cases, the origin was unclear. All patients had been evaluated by cranial, thoracic, and abdominal computed tomography (CT). It is noteworthy that in six patients, bacterial sepsis preceded mucormycosis; four of them had already been admitted to the intensive care unit prior to mucormycosis diagnosis.

**Figure 1:**
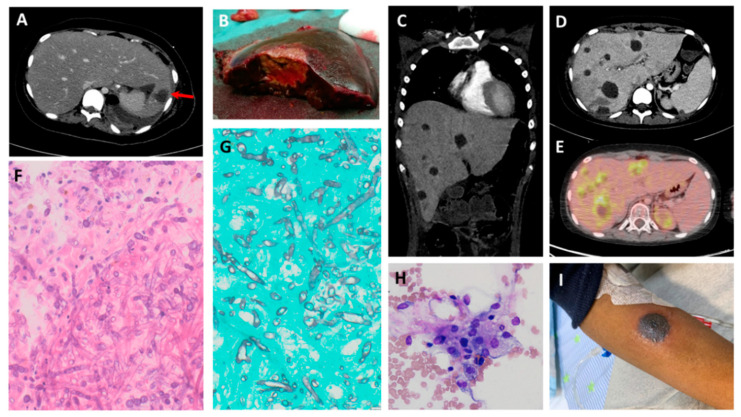
Typical radiographic and histopathological features of disseminated mucormycosis. (**A**) Axial abdominal CT of a 13.5-year old girl with ALL, demonstrating a hypodense lesion in the left hepatic lobe (red arrow). Additional sites of involvement include the spleen, left kidney, bowel, and abdominal wall (not shown) (**B**) A large fungal abscess in the resected left hepatic lobe of the girl in (**A**). (**C**) Coronal chest and abdominal CT scans of a 14.5-year old girl with AML, showing multiple hypodense hepatic lesions, with additional bowel involvement. (**D**) Axial abdominal CT scan of the girl in (**C**), demonstrating multiple hepatic lesions. (**E**) Axial abdominal PET-CT scan of the girl in (**C**), showing multiple fluorodeoxyglucose (FDG)-avid hepatic lesions, despite three months of antifungal therapy. (**F**) Biopsy of a hepatic lesion from (**C**), demonstrating numerous large fungal hyphae with minimal septation and branching (hematoxylin and eosin, 400×). (**G**) The hyphal elements in the hepatic lesion are highlighted by Grocott methenamine silver stain (GMS; 400×). (**H**) Due to prolonged severe pancytopenia, a bone marrow aspiration was performed, disclosing prominent hemophagocytosis (hematoxylin and eosin, 100×). (**I**) One of several necrotic cutaneous lesions in a 13.5-year old boy with AML.

All cases were classified as proven by EORTC/MSG criteria, with evidence by pathology (*n* = 11) and/or culture (*n* = 7). In addition, in seven cases, the pathogens were molecularly confirmed by PCR. Isolates included *Rhizopus* (*n* = 4), *Mucor* (*n* = 2), and *Lichtheimia* species (*n* = 4). In two cases, the specific pathogen was unknown (Table 1). Antifungal susceptibility testing was performed in four cases (Table 2). One patient (patient no. 5) was diagnosed post-mortem. Ten patients received prophylactic antifungal therapy, but none with Mucorales-active agents (Table 3). In four cases, fungal co-infections (proven/probable) with candida (*n* = 1) or aspergillus (*n* = 3) were diagnosed in addition to mucormycosis. The radiographic and histopathological features are shown in Figure 1.

### 3.3. Treatment

**Figure 2:**
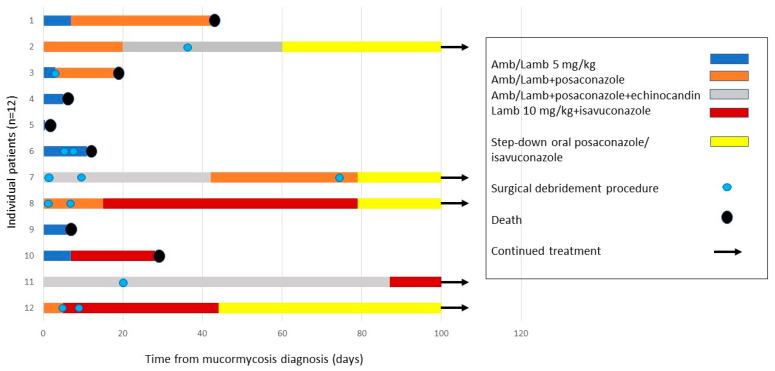
Treatments and outcomes of 12 immunocompromised children with disseminated mucormycosis. Swimmers plot illustrating the treatment modalities and survival of the 12 individual patients in this study.

The treatment modalities are summarized in Table 3 and Figure 2. In all cases, amphotericin B (AmB; *n* = 4) or liposomal amphotericin (L-AmB; *n* = 8) were administered as first-line therapy. Of the eight cases treated with liposomal amphotericin, three were treated at a dose of 5 mg/kg/day, and five with higher doses of 7.5–10 mg/kg/day. In eight cases, combination antifungal therapy, comprising of AmB/L-AmB along with an echinocandin (caspofungin or anidulafungin) or triazole (posaconazole or isavuconazole), was administered as first-line or salvage therapy. All survivors were treated with prolonged step-down therapy.

Seven of the twelve patients underwent surgical debridement procedures, mainly bowel resections and the excision of visceral organs (nephrectomy, splenectomy, or partial hepatectomy). Pulmonary lobectomy and functional endoscopic sinus surgeries (FESS) were also performed.

Adjunctive therapies included granulocyte colony-stimulating factor (G-CSF) in nine patients and granulocyte infusions in five. Two patients with ALL were rapidly weaned from corticosteroid therapy. In one patient, after liver transplantation, immunosuppression was stopped. A patient with AML (patient no. 11) had a high fever and persistent severe pancytopenia, despite G-CSF therapy. Bone marrow aspiration on day 14 from mucormycosis diagnosis demonstrated prominent hemophagocytosis (Figure 1H). Blood tests disclosed elevated levels of ferritin (57,396 ng/mL), soluble IL2 receptor (10,071 units/mL), and triglycerides (400 mg/dL), suggesting a diagnosis of secondary hemophagocytic histiocytosis. She was treated with 10 mg/m^2^ of dexamethasone, with subsequent blood count recovery within several days. 

### 3.4. Outcome 

**Figure 3:**
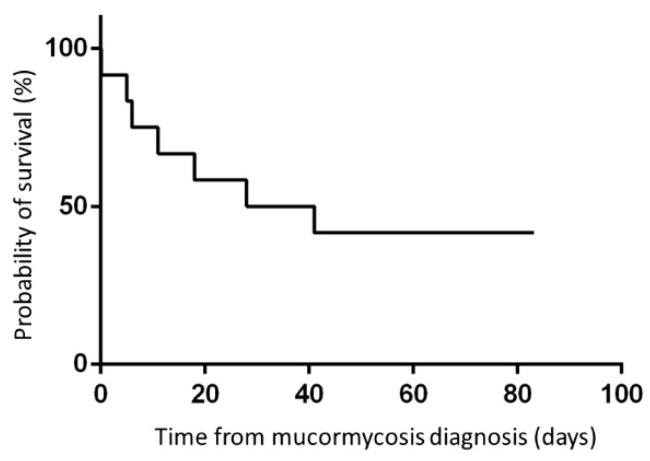
Outcome: 6-week probability of survival of the 12 study patients: 42%.

Seven patients died with active mucormycosis, all within six weeks of diagnosis (case fatality rate: 58%; Figure 2 and Figure 3). The median time to death was 11 days. Of the six patients diagnosed between 2009–2015, only one survived. Four of the six patients diagnosed between 2016–2020 were salvaged. The five patients who survived mucormycosis are currently alive; four patients are in complete remission from their underlying malignant diseases, and one liver transplant recipient has a functioning liver allograft (median follow-up: 41.4 months).

Two of the five survivors had already completed treatment for their underlying conditions at the time of mucormycosis diagnosis. An 11-year old boy (patient no. 7) was diagnosed with mucormycosis following four autologous HSCTs for the treatment of metastatic medulloblastoma. He was treated with an L-AmB/echinocandin combination, followed by L-AmB/intravenous posaconazole, in addition to bowel resections, two partial hepatectomies, and extensive debridement procedures. He received oral posaconazole step-down treatment for 9.5 months from the mucormycosis diagnosis. A 13.5-year old boy (patient no. 12) was diagnosed with disseminated skin mucormycosis after a final course of AML therapy (Figure 1I). He was treated with combined L-AmB/intravenous posaconazole, followed by L-AmB/ isavuconazole. Skin lesions were surgically excised, and a pulmonary lobectomy revealed co-infection with *Aspergillus niger*. Currently, eight months since mucormycosis diagnosis, he is continuing treatment with step-down oral isavuconazole.

In three of the five survivors, treatment of the underlying conditions was continued as soon as mucormycosis was deemed to be under control. A 10-year old girl, after liver transplantation (patient no. 2), successfully underwent liver re-transplantation, along with the re-institution of immunosuppression. She continued oral posaconazole for 12 months thereafter. A 13.5-year old girl (patient no. 8) was diagnosed with mucormycosis during induction for Philadelphia-positive ALL. She underwent two extensive surgical procedures, with multiple bowel and visceral resections (Figure 1A,B) and a lengthy treatment with combined L-AmB/isavuconazole. Because of an inability to tolerate chemotherapy, she continued her leukemia therapy with dasatinib, a tyrosine kinase inhibitor, and blinatumomab, an immunotherapeutic agent. Prolonged step-down posaconazole therapy was administered until the end of intensive leukemia therapy (12 months after mucormycosis diagnosis), at reduced doses as a result of a pharmacokinetic interaction with dasatinib. The third survivor, a 14.5-year old girl (patient no. 11), developed mucormycosis after three courses of chemotherapy, while in remission from AML. She was first treated with combined L-AmB/intravenous posaconazol, and later, because of the persistence of inoperable widespread hepatic mucormycosis (Figure 1C,D), treatment was changed to combined L-AmB/isavuconazole. She underwent bowel resections and extensive intra-abdominal debridement with improvement, but with persistent active hepatic lesions shown upon positron emission tomography-computed tomography (PET-CT; Figure 1E), confirmed by biopsy (Figure 1F,G). At 118 days from mucormycosis diagnosis, she was diagnosed with AML relapse. On day 138, she underwent an allogeneic stem cell transplantation from a matched sibling donor, with alpha–beta T-cell depletion and a conditioning regime consisting of fludarabine, melphalan, and thiotepa. During transplantation, she was consistently treated with combined L-AmB/isavuconazole, and with daily granulocyte infusions. Currently, it has been one year from her mucormycosis diagnosis and 220 days since her HSCT. She continues treatment with oral posaconazole. Her PET-CT still demonstrates stable hepatic lesions, with pathological fluorodeoxyglucose (FDG) uptake.

## 4. Discussion

In this multicenter study, we report twelve children diagnosed with disseminated mucormycosis at six tertiary medical centers in Israel over a period of eleven years. Of the twelve patients in our study cohort, seven died with active mucormycosis within six weeks. Among the patients diagnosed between 2009–2015, one of the six patients survived, and of those diagnosed between 2016–2020, four of the six patients were salvaged.

All twelve patients in our study were treated with amphotericin B products as first-line therapy, with a gradual transition from AmB to L-AmB over the years, in accordance with the current recommendations [7,8,9]. The benefit of higher doses of L-AmB (5–10 mg/kg/day), as administered to five of the study patients (four of them survivors), is yet unproven. Dose-dependent efficacy has been demonstrated in vitro and in animal models [19], but not in clinical studies [20]. Higher doses (5–10 mg/kg/day) are currently recommended for CNS involvement [7]. 

Treatment of invasive fungal infections has evolved considerably in recent years with the introduction of new azoles, posaconazole and isavuconazole, to the therapeutic armamentarium. Because of concerns regarding the variable bioavailability of posaconazole oral suspension, more stable delayed-release tablets and an intravenous formulation have recently been made available. Isavuconazole, intravenous posaconazole, and delayed release posaconazole tablets are currently recommended for salvage therapy in cases of refractory mucormycosis or intolerance of first-line antifungal agents, and are moderately recommended as first-line therapy [7,21]. These antifungal agents have been in use among our study patients in Israel since 2016. 

Seven of the twelve patients with disseminated mucormycosis in our study received combination therapy consisting of AmB/L-AmB with an echinocandin (caspofungin) or triazole (posaconazole/isavuconazole). In view of the high mortality rates of mucormycosis, combined antifungal therapy seems an attractive option. A possible benefit has been shown in some murine models and patient series, but its efficacy has not been demonstrated in a clinical study [22]. In this retrospective study, all five survivors received combined L-AmB with triazole therapy, and in three cases, they also received isavuconazole. Two exhibited a notable improvement following the addition of isavuconzole.

However, it is difficult to attribute improved salvage rates to pharmacological factors alone [23]. Nonpharmacologic parameters, such as the timing of diagnosis, onset of antifungal therapy [24], recovery from immunosuppression, and feasibility and extent of surgical procedures, have a critical impact upon outcome. 

Rapid diagnosis is a significant challenge in disseminated mucormycosis, as a result of the nonspecific nature of the clinical presentation. Most of our patients had prolonged neutropenic fever. Several were otherwise asymptomatic and were diagnosed following empirical imaging studies. Gastrointestinal involvement occurred in six of the twelve patients in our study group. Patients with gastrointestinal involvement are characterized by a clinical presentation that typically resembles neutropenic enterocolitis, with a rapid progression and imminent risk of gastrointestinal perforation. Sudden, unexplained bowel necrosis in neutropenic patients with hematological malignancies should arouse suspicion of mucormycosis, and the microbiology laboratory should be urgently alerted. In this study, cultures were positive for mucormycosis in only seven of the twelve cases, and molecular tests performed on fresh clinical specimens in the majority of cases significantly contributed to a swift verification of the diagnosis [9]. Further advances in molecular strategies hold promise for a more timely diagnosis [25]. A high index of suspicion should be maintained when treating children with hematological malignancies. 

Surgical interventions are considered a cornerstone of mucormycosis management, and current guidelines strongly recommend early complete surgical debridement procedures [7]. In our study, seven of the twelve patients underwent surgical debridement procedures, including all five survivors. Several studies have reported improved survival rates for patients who underwent surgical interventions [26], although possible selection bias must be taken into account.

The role of surgical interventions has been firmly established in mucormycosis management. The benefits of repeated extensive debridement with clean margins have been shown in rhino-orbito-cerebral mucormycosis [27], as well as in skin and soft-tissue infections [28]. Thus, the concept of surgical debridement is understandable for local control, so as to prevent dissemination. However, the role of surgical debulking in the management of disseminated disease has not yet been determined. This issue is especially significant when facing a critically-ill patient in whom complete debridement of disseminated disease is not feasible. Surgical procedures may prevent life-threatening complications. For example, bowel resection of involved areas may prevent intestinal perforations and lung resections may prevent massive bleeding episodes [29]. In addition, mucormycosis infections are characterized by extensive angioinvasion, with vessel thrombosis and subsequent tissue necrosis, which may impair antifungal agent penetration and bioavailability, as well as prevent the delivery of immune cells [30]. Surgical debulking via visceral excision may facilitate the clearance of infection from sites that are inaccessible to antifungal agents.

In our study, four of the five survivors had hepatic involvement (Figure 1A–H). One underwent a liver allograft resection with subsequent re-transplantation; two underwent partial hepatectomy with the resection of mucormycosis abscesses; and in the fourth case, the multiple hepatic lesions were inoperable—yet all four patients survived. Remarkably, the fourth survivor proceeded to HSCT, while still harboring multiple FDG-avid hepatic lesions, confirmed by biopsy as active mucormycosis. The true impact of visceral excision on the outcome of disseminated mucormycosis is difficult to elucidate. 

The reversal of immunosuppression is of paramount importance in the management of mucormycosis, and underlying conditions significantly impact outcome. In developed countries, the most common underlying conditions in patients with mucormycosis are hematological malignancies and HSCT. Dissemination reportedly occurs more frequently in these severely immunosuppressed patients. In this study, none of the patients had an uncontrolled, refractory hematological malignancy; in such cases, the reversal of immunosuppression is virtually impossible, leading to a poor outcome [3]. The two patients who did not have an underlying hematological malignancy in our study were among the survivors. Another factor that may affect outcome is disease localization. For example, in this study, one patient with a disseminated skin infection survived, but all patients with CNS involvement (*n* = 4) did not.

There are a lack of data from controlled clinical trials to guide the management of mucormycosis. Disseminated mucormycosis is a rare entity, with limited data regarding the outcome and management, especially in children. Our population-based study, despite its limited number of patients, highlights several important issues concerning the treatments and outcomes of children with this entity. In our study cohort, we note improved salvage rates in recent years, concurrent with the availability of new antifungal agents. Other factors that may have contributed to an improved outcome are patients’ underlying conditions, specific disease localizations, and a heightened index of suspicion.

## 5. Conclusions

In this multicenter population-based study, we report twelve children diagnosed with disseminated mucormycosis over a period of eleven years. Disseminated mucormycosis is a life-threatening and often fatal disease, and improved diagnostic and therapeutic strategies are needed. Nevertheless, in our study, five patients were salvaged, most of them in recent years, through combined L-AmB/triazole antifungal therapy and extensive surgical interventions. 

## Figures and Tables

**Table 1:** Patient demographics and microbiological data in 12 pediatric patients with disseminated mucormycosis.

	Sex	Age (Years)	Underlying Condition	Phase of Therapy	Duration of Neutropenia (Days)	Clinical Presentation	Sites of Involvement	Pathogen	Diagnostic Method	Preceding Bacterial Infection (within 14 Days)	Fungal Co-Infection
1	M	19.2	Relapsed ALL	Post-allogeneic HSCT (19 days)	21	Pleuritic chest pain	Lung, skin, and soft tissue	*Rhizopus oryzae*	Culture		
2	F	10	SOT—liver	Post liver-transplant (8 weeks)	0	Prolonged fever	Liver allograft, pericardial effusion, diaphragm, and gastric wall	*Rhizopus oryzae*	Pathology and PCR	E. coli peritonitis	*Candida krusei* peritonitis (proven)
3	M	8.9	ALL	Delayed intensification	8	Prolonged fever and obtundation	Lung, sinus, and brain	*Rhizopus oryzae*	Pathology and PCR		
4	F	16.5	AML	Consolidation	14	Prolonged fever and convulsions	Lung, brain, liver, and kidneys	*Rhizopus pusillus*	Pathology and PCR	K. pneumoniae sepsis	
5	F	16	ALL	Induction	35	Hemiparesis and convulsions	Lung, heart, bowel, brain, kidneys, and liver	*Mucor spp*	Pathology and culture	S. aureus sepsis	
6	M	11.5	ALL	Induction	22	Fever and abdominal pain	Bowel, liver, spleen, and gastric wall	Unknown	Pathology		
7	M	11.2	Metastatic medulloblastoma post-autologous HSCT	Post- fourth autologous HSCT (10 days)	8	Fever and abdominal pain	Bowel, liver, abdominal abscesses, and lung	*Lichtheimia ramosa*	Culture, pathology, and PCR		
8	F	13.4	ALL	Induction	25	Prolonged fever and signs of peritoneal irritation	Liver, spleen, bowel, and kidney	*Lichtheimia corymbifera*	Culture, pathology, and PCR	P. aeruginosa sepsis	Pulmonary aspergillosis (probable)
9	F	15	AML	Induction	28	Acute renal failure requiring hemodialysis	Brain, lung, bowel, kidneys, liver, and spleen	Unknown	Pathology	A. baumani sepsis	
10	F	10	Mature B-cell (Burkitt) leukemia	Induction	11	Fever and abdominal pain	Sinus, liver, and bowel	*Lichtheimia ramosa*	Culture and pathology	A lwoffii sepsis	Pulmonary aspergillosis (probable)
11	F	14.4	AML	Consolidation	11	Fever and abdominal pain	Liver, bowel, and lung	*Mucor spp.*	Culture, pathology, and PCR	K pneumoniae sepsis	
12	M	13.5	AML	Consolidation	7	Necrotic cutaneous lesions	Disseminated skin (right arm, right foot, right ankle, and scalp)	*Lichtheimia ramosa*	Culture, pathology, and PCR		Pulmonary aspergillosis (*Aspergillus niger)* (proven)

ALL—acute lymphoblastic leukemia; AML—acute myeloid leukemia; HSCT—hematopoietic stem cell transplantation; PCR—polymerase chain reaction; SOT—solid organ transplantation.

**Table 2:** Antifungal susceptibility testing in four cases: minimal inhibitory concentrations (MICs; mg/L).

		Amphotericin B	Posaconazole	Voriconazole	Itraconazole	Caspofungin	Anidulafungin
Patient no. 8	*Lichtheimia corymbifera*	0.125	0.5	0.5	0.25	>8	>4
Patient no. 10	*Lichtheimia ramosa*	0.25	2	>16	>16	>8	2
Patient no. 11	*Mucor spp.*	0.06	1	>16	>16	>8	>16
Patient no. 12	*Lichtheimia ramosa*	0.125	1	>16	2	>5	>16

**Table 3:** Treatments and outcomes of 12 pediatric patients with disseminated mucormycosis.

	Year of Mucormycosis Diagnosis	Antifungal Prophylaxis	First-Line Antifungal Treatment	Second-Line Antifungal Treatment	Step-Down Treatment	Surgical Debridement Procedures	Outcome	Follow-Up (Days)
1	2009	Voricinazole	AmB	L-AmB 5 mg/kg/d posaconazole susp		None	Death	42
2	2009	Fluconazole	L-AmB 5 mg/kg/d posaconazole susp	L-AmB 5 mg/kg/d caspofunginposaconazole susp	Posaconazole susp (12 months)	Liver re-transplantation, splenectomy, partial gastrectomy, thoracotomy, and pericardial window	Survival	4188 (137.5 months)
3	2010	None	AmB caspofungin	L-AmB 5 mg/kg/d posaconazol susp		FESS	Death	18
4	2012	Itraconazole	AmB	L-AmB 5 mg/kg/d		None	Death	5
5	2012	Fluconazole	L-AmB 5 mg/kg/d	-		None	Death	0
6	2015	Itraconazole	AmB	-		1. Resection of transverse colon 2. Resection of small intestine 3. Exploratory laparotomy	Death	11
7	2016	Fluconazole	L-AmB 7.5 mg/kg anidulafungin/ caspofungin	L-AmB IV posaconazole	PO posaconazole TAB	1. Appendectomy and drainage of hepatic abscess 2. Right hemicolectomy, partial hepatectomy, debridement of diaphragm and abdominal wall, and thoracoscopic decortication of right lung =3. Repeat partial hepatectomy	Survival	1797 (59 months)
8	2017	Itraconazole	L-AmB 5 mg/kg/d IV posaconazole	L-AmB 10 mg/kg/d IV isavuconazole	PO isavuconazole, followed byposaconazole TAB	1. Bowel resections 2. Splenectomy, partial hepatectomy, left nephrectomy, and debridement of abdominal wall	Survival	1260 (41.4 months)
9	2019	Fluconazole	L-AmB 5mg/kg/d	-		None	Death	6
10	2019	Anidulafungin	L-AmB 5mg/kg/d	L-AmB 10 mg/kg/d IV isavuconazole		None	Death	28
11	2020	Itraconazole	L-AmB 10 mg/kg/d IV posaconazole caspofungin	L-AmB 10 mg/kg/d IV isavuconazole	Posaconazole TAB	Right hemicolectomy, left hemicolectomy, partial resection of small intestine, partial hepatectomy, and omentectomy	Survival	363 (11.9 months)
12	2020	None	L-AmB 5 mg/kg/d IV posaconazole	L-AmB 7.5 mg/kg IV isavuconazole	PO isavuconazole	1. Excisions of skin lesions 2. FESS 3. Pulmonary left lower lobe lobectomy	Survival	261 days (8.6 months)

AmB—amphotericin B; FESS—functional endoscopic sinus surgery; L-AmB—liposomal amphotericin; susp—oral suspension; TAB—tablets.

## Data Availability

Deidentified data presented in this study are available on request from the corresponding author.
